# Cefepime-Induced Mixed Hepatocellular and Cholestatic Liver Injury: A Case Report

**DOI:** 10.7759/cureus.73393

**Published:** 2024-11-10

**Authors:** Mahyar Toofantabrizi, Anuj Timshina, Raj M Dongol

**Affiliations:** 1 Internal Medicine, MedStar Union Memorial Hospital, Baltimore, USA; 2 Internal Medicine, MedStar Franklin Square Medical Center, Baltimore, USA

**Keywords:** cefepime, cholestatic injury, drug-induced liver injury (dili), hepatocellular injury, idiosyncratic

## Abstract

Drug-induced liver injury (DILI) presents significant diagnostic challenges, particularly in patients with multiple comorbidities. We report a case involving a 72-year-old female treated with cefepime for urosepsis, who developed markedly elevated liver enzymes after two weeks of therapy. After excluding other potential causes, including viral hepatitis, ischemia, and autoimmune hepatitis, cefepime-induced mixed pattern liver injury was determined to be the likely etiology of the elevated liver enzymes. This case underscores the importance of considering DILI in the differential diagnosis and emphasizes the necessity for vigilant monitoring and early recognition, particularly in elderly patients.

## Introduction

Drug-induced liver injury (DILI) is a common condition that can arise from nearly all classes of medications, with an annual incidence in the general population of approximately 15-20 per 100,000 in the United States and worldwide. However, its true incidence is difficult to ascertain, as it is frequently underreported [1,2]. DILI is the leading cause of acute liver failure in the United States [3], and antibiotic-induced hepatotoxicity accounts for 25-45% of DILI cases [3]. While cephalosporins are generally well-tolerated and associated with minimal adverse effects, they account for about 1% of DILI cases in prospective studies [4,5]. Cefepime, a fourth-generation cephalosporin antibiotic with a broad spectrum of bacterial activity, is widely used in hospital settings and is very rarely linked to liver injury. A review of the literature reveals only four reported cases of DILI associated with cefepime administration, including one large retrospective study, one large prospective study, and two individual case reports [6-9].

DILI can be a serious and potentially life-threatening condition [10]. Mortality rates from DILI with jaundice related to antibiotics have been reported at approximately 10%, with some patients requiring liver transplantation due to acute fulminant hepatic failure [1,11]. There are two primary types of DILI: (1) idiosyncratic DILI, which is unpredictable and independent of drug dosage, typically presenting with a latency period of seven to 14 days and (2) direct DILI, which is more predictable and dose-related. Paracetamol (acetaminophen) poisoning is the most common cause of direct DILI, accounting for nearly half of all acute liver failure cases [12]. Paracetamol hepatotoxicity occurs through the formation of the toxic metabolite N-acetyl-p-benzoquinone imine, which depletes adenosine triphosphate levels by binding to mitochondrial proteins [13]. The precise mechanisms underlying idiosyncratic DILI remain unclear, although they may be immune or non-immune-mediated. Factors contributing to idiosyncratic DILI include drug-related factors such as dose, duration, and lipophilicity; host-related factors such as age, gender, genetic polymorphisms, and immune status; and environmental factors such as concomitant alcohol use, diet, and tobacco [14]. Specific genetic factors, such as certain human leukocyte antigen (HLA) types, are often associated with an increased risk of idiosyncratic DILI. For instance, the HLA-A02:01 and HLA-DRB1*15:01 alleles have been linked to a higher risk of DILI with amoxicillin-clavulanate, supporting the hypothesis that the drugs or their metabolites bind to the T-cell receptor groove influenced by the associated HLA [15,16].

As the liver ages, its capacity to recover from injury diminishes, making older individuals more susceptible to DILI [17]. The presence of comorbidities and polypharmacy, which frequently accompany older age, is also thought to contribute to this risk [18]. Approximately 20% of patients with DILI may develop chronic liver injury, manifesting as persistent inflammation or diminishing bile ducts, potentially progressing to cirrhosis and necessitating liver transplantation [19]. In this case, we report a 72-year-old female who was treated with cefepime for urosepsis and subsequently presented with a liver injury two weeks after the initiation of therapy.

## Case presentation

A 72-year-old female with a history of insulin-dependent type 2 diabetes, hypertension, hypothyroidism, chronic kidney disease (CKD) stage 4, anxiety, incomplete bladder emptying/voiding dysfunction with a chronic indwelling Foley catheter, and breast cancer status post bilateral mastectomy was referred to the emergency department by her primary care physician due to markedly elevated liver enzymes. Despite these alarming lab results, the patient remained largely asymptomatic, except for fatigue. She denied any acute symptoms such as nausea, vomiting, abdominal pain, or jaundice, and also denied alcohol or any recreational drug use.

Her past medical history included a recent hospitalization two weeks prior for urosepsis, hyperosmolar hyperglycemic state, type 2 non-ST elevation myocardial infarction, and acute kidney injury on CKD. During that admission, she was also on simvastatin 40 mg daily, which she had been taking for three years, in addition to the medications listed above. At that time, her liver enzymes were slightly elevated, but the cause was attributed to dehydration and urosepsis. Simvastatin was held on the second day of admission due to the risk of hepatotoxicity. Dehydration was corrected, and sepsis was treated with intravenous cefepime. Liver enzymes began to trend downward from the third day of admission, and the patient was discharged home after five days, with simvastatin on hold at discharge.

Upon her next presentation, about two weeks after the previous admission, her liver function tests revealed significantly elevated levels, with aspartate aminotransferase at 2,932 units per liter and alanine aminotransferase (ALT) at 1,112 units per liter. Imaging studies, including a CT scan of the abdomen and pelvis and a right upper quadrant ultrasound, showed no significant liver or biliary pathology. Differential diagnoses considered included viral hepatitis, ischemia, autoimmune hepatitis, and DILI. Acetaminophen and salicylate levels were low, and the viral panel was negative for hepatitis A, B, and C. Ischemia was deemed unlikely due to stable vital signs and no recent history of shock or surgery. Autoimmune hepatitis also appeared unlikely given the patient’s age and lack of autoimmune history or symptoms.

A review of recent medications highlighted a five-day course of intravenous cefepime at 1 gram twice daily for sepsis due to a urinary tract infection during her recent admission. Literature suggests that cefepime, although rarely, can cause mixed liver injury, which aligns with this patient’s clinical picture. DILI typically presents as an acute illness characterized predominantly by fatigue, nausea, malaise, pruritus, and jaundice, which generally resolve quickly after stopping the offending agent. Given the exclusion of other causes and the timeline of liver enzyme elevation correlating with cefepime use, it is likely that cefepime-induced liver injury was responsible for this patient’s DILI. The Rousse Uclaf Causality Assessment Method of the Council of International Organization of Medical Science (RUCAM/CIOMS), the most frequently utilized criteria set for diagnosing DILI, was applied in this case. The RUCAM score was determined to be 6, indicating cefepime as the probable cause of the liver injury. Liver panel tests showed improvement during hospitalization (Table 1, Table 2).

**Table 1:** Serial liver enzymes during prior admission ALP, alkaline phosphatase; ALT, alanine aminotransferase; AST, aspartate aminotransferase

Liver enzyme (unit/liter)	Normal range	Day 1 (8/7/24)	Day 2 (8/8/24)	Day 3 (8/9/24)	Day 4 (8/10/24)	Day 5 (8/11/24)
AST	0-33	224	224	91	81	29
ALT	10-49	494	494	307	276	143
ALP	46-116	354	355	286	273	209

**Table 2:** Serial liver enzymes during recent admission ALP, alkaline phosphatase; ALT, alanine aminotransferase; AST, aspartate aminotransferase

Liver enzyme (unit/liter)	Normal range	Day 1 (8/22/24)	Day 2 (8/23/24)	Day 3 (8/24/24)
AST	0-33	2,932	1,183	768
ALT	10-49	1,112	783	649
ALP	46-116	456	378	335

## Discussion

Accurate diagnosis of DILI necessitates a comprehensive evaluation, including detailed historical and laboratory data. Key steps in this process involve noting the latency of symptom onset, assessing the rate of resolution following medication discontinuation, excluding other potential causes of liver injury, and comparing clinical features to established patterns associated with the suspected drug [20]. Acute viral hepatitis must also be considered in the differential diagnosis, prompting serological tests for hepatitis A, B, C, and E, as well as screening for cytomegalovirus, Epstein-Barr virus, herpes simplex virus, and varicella zoster virus if other possibilities are ruled out.

DILI is categorized as hepatocellular, cholestatic, or mixed, based on the “R-value,” which is the ratio of serum ALT to alkaline phosphatase (ALP) activity (Table 3) [20]. In our patient’s case, the R-value was 2.4 (1,112/456), with elevated ALP indicating a mixed pattern of liver injury. However, an ALT elevation of three times above the upper limit of normal is not always sufficient to identify clinically relevant DILI, as certain medications, such as antitubercular drugs, can cause an increase in aminotransferase levels that may subside despite ongoing treatment - a phenomenon known as adaptation, which does not necessarily indicate significant liver damage [21].

**Table 3:** Types of DILI R-value = ratio of serum ALT to ALP [20] Alk Phos, alkaline phosphatase; ALP, alkaline phosphatase; ALT, alanine aminotransferase; DILI, drug-induced liver injury; ULN, upper limit of normal

Type	Enzymatic profile	Prognosis
Hepatocellular	ALT > 3ULN, R ≥ 5	More severe prognosis
Cholestatic	Alk Phos ≥ 2ULN, R ≤ 2	More prone to chronic disease
Mixed	ALT > 2 ULN R between 2 and 5	More prone to chronic disease

Mitchell et al. (1975) conducted a study involving patients in a psychiatric hospital during a tuberculosis outbreak treated with isoniazid (INH) and observed clinical adaptation. They monitored liver enzymes every four weeks, collecting blood samples before and after treatment. The results revealed that 38% of patients experienced elevated liver enzymes, yet most cases resolved despite the continued use of INH [22]. Additionally, hepatic enzyme levels can temporarily rise in patients with acute gastroenteritis due to severe dehydration, but these levels typically normalize after fluid repletion. In our patient’s case, the elevation in liver function tests during her prior admission was attributed to her infection and dehydration.

As previously mentioned, idiosyncratic DILI is unpredictable, non-reproducible, and occurs in susceptible individuals, usually independent of drug dosage. It typically has a latency period of seven to 14 days and can manifest with symptoms ranging from mild liver enzyme elevations to acute liver failure. Common symptoms resemble those of hepatitis, including nausea, jaundice, fatigue, and sometimes right upper quadrant pain [3,12]. However, our patient denied experiencing any of these symptoms and remained asymptomatic, apart from fatigue. Idiosyncratic DILI can affect individuals of all ages, with hospitalization required in 23% of cases. This accounts for 3.5% of hospital admissions for jaundice and 11% of acute liver failure cases in the United States [21,23,24]. Our patient is a 72-year-old female with multiple comorbidities, which aligns with the findings in the literature. She received a renal-adjusted dose of cefepime during her previous hospital stay. Notably, she did not receive any other medications known to cause liver injury, nor any drugs that could interact with those causing liver injury. Her statin medication was discontinued on the second day of her prior admission, during which her liver enzymes were elevated but subsequently improved with the correction of dehydration and treatment of sepsis. However, her liver enzymes became significantly elevated after 14 days, although the exact timing of this increase is unclear due to the absence of interim enzyme level monitoring.

Cefepime, a fourth-generation cephalosporin with broad-spectrum activity, is generally considered safe with minimal side effects. Although cefepime has been associated with rare cases of neurotoxicity, encephalopathy, and acute kidney injury, reports of DILI remain very rare, and the exact mechanism of injury is still unclear but may be mediated through immunologic reactions [25,26].

Tests for anti-neutrophil antibody (ANA) and anti-mitochondrial antibody (AMA) can aid in the diagnosis of DILI, with studies indicating that ANA titers are more specific for DILI caused by certain medications [27]. In contrast, AMA positivity is linked to more severe liver damage than ANA. Our patient had negative ANA and AMA levels. Various studies suggest that monocyte-derived hepatocyte-like cell tests can be a novel tool for identifying DILI with higher specificity; however, this test was not performed for our patient [28]. A systematic review and meta-analysis by Li et al. reported an overall incidence of DILI at 4.94 per 100,000 person-years, with Asia having the highest incidence at 17.82 and North America the lowest at 1.72 per 100,000 person-years [29].

Regarding management, there is no specific protocol to follow. The primary approach involves discontinuing the offending agent, avoiding hepatotoxic substances, and providing supportive care. However, for certain types of direct DILI, an antidote may be available and should be administered when appropriate.

## Conclusions

This case underscores the diagnostic challenges associated with DILI. Although cefepime is generally well tolerated, it can lead to DILI, particularly in older adults with multiple comorbidities. The case emphasizes the importance of vigilant liver function monitoring in patients receiving cefepime, as well as the need for early recognition of DILI to prevent chronic liver damage and potentially life-threatening fulminant liver failure. Discontinuing cefepime and providing early supportive care are critical interventions if DILI is suspected. Given that patients may present with symptoms as late as two weeks after initiating cefepime, as demonstrated in this case, it is essential to provide thorough counseling regarding the risk of DILI and its associated symptoms, which can arise even after discharge.
